# Assessment of Healing Potential of Alginate and Chitosan‐Based Biomaterials for Cranial Bone Defects in Experimental Model

**DOI:** 10.1155/bmri/3257416

**Published:** 2026-04-15

**Authors:** Oleksii V. Korenkov, Liudmyla B. Sukhodub, Mariia O. Kumeda, Leonid F. Sukhodub

**Affiliations:** ^1^ Laboratory of Bionanocomposite, Sumy State University, Sumy, Ukraine, sumdu.edu.ua

**Keywords:** alginate, chitosan, computed tomography, HPLC study, skull vault regeneration

## Abstract

The healing of bone defects in the skull vault is a serious clinical problem. The aim of this study is to evaluate the effect of two osteoplastic materials on the healing of a cranial vault experimental defect by computed tomography. Two material systems are analyzed in detail: (i) a sodium alginate–based matrix modified with hydroxyapatite microparticles and zinc ions and (ii) a chitosan‐based matrix containing in situ–formed brushite nanoparticles; both sample types were loaded with cholecalciferol (VitD3). A total of 36 rats aged 6 months showed that the alginate‐based biomaterial promotes complete healing of the parietal bone defect after 200 days, whereas healing with the chitosan‐based biomaterials at that time was not complete. The second objective was to determine the kinetics of VitD3 release from experimental biocomposites using high‐performance liquid chromatography. The kinetics of VitD3 release depends on the material′s composition: The composite with chitosan prolongs the drug release to 48 h without the so‐called “burst release.” In the case of the alginate composite, a “burst release” of vitamin D3 is observed during the first 4 h. Both osteoplastic materials show the potential to optimize reparative osteogenesis in an experimental defect of the flat skull bone, although the regenerative potential of the alginate‐based biomaterial is higher.

## 1. Introduction

The restoration of bone defects in the cranial vault remains a serious clinical problem today [1]. Many bone grafting operations are performed annually worldwide, with high complications and economic costs [2, 3]. Congenital malformations of the skull, injuries, surgical operations, tumor resection, and infections can all lead to the formation of a defect in flat bones [4, 5]. At the same time, numerous experimental works have proven that the intramembranous bones of the cranial vault under normal conditions have weak regenerative capabilities, which depend on the species of animals, the origin of the bones, and the location and size of the defect. For example, it has been established that in adult dogs, a trepanation defect of the cranial vault measuring 1.4 cm does not heal throughout life [6], and a 22‐mm defect is filled with only 16.1*%* ± 10.6*%* of regenerated bone tissue in 16 weeks [7]. In rabbits, a 10‐mm diameter defect in the parietal bone forms 18% of regenerated bone tissue after 12 weeks, and 90 days after the application of an 8‐mm diameter defect, clear boundaries of the injury site with low radiopacity are determined [7, 8]. In young and mature mice, bone defects originating from the neural crest (frontal) heal better than bones formed from paraxial mesoderm (parietal) [9]. The regeneration of the cranial vault bones also depends on the distance between the injury site and the craniocerebral sutures. It is known that bone defects that are located closer to the suture heal better [10]. Mechanical loads acting on the injured bone (in the bones of the cranial vault, it is minimal) and the dura mater, without the integrity of which bone cannot be restored, play a significant role in the healing of bone defects [11–15]. In addition, the animal′s age plays a leading role in the regenerative ability of the flat bones of the skull. For example, parietal bone defects measuring 3, 4, and 5 mm in 60‐day‐old mice were filled mainly with loose connective tissue (bone tissue was less than 5%) within 8 weeks. In 6‐day‐old mice, almost complete closure of the defect with regenerated bone tissue occurred over the same period [16]. One example of the molecular mechanisms that allow the bones of the cranial vault to heal after injury in 6‐day‐old mice is the significantly higher expression of molecular factors such as bone morphogenetic proteins‐2, 4, and 7 (BMP‐2, ‐4, and ‐7), insulin‐like growth factor‐2 (IGF‐2), pleiotrophin (PTN), fibroblast growth factor‐2 (FGF‐2), Fibroblast growth factor receptor‐1 (FGFR‐1), osteonectin, osteocalcin, tartrate‐resistant acid phosphatase (Acp5), cathepsin K (Ctsk), and matrix metallopeptidase 2 (Mmp2) in the regenerated parietal bones compared with adult mice [17]. The importance of FGF‐2 and BMP‐2 expression is also indicated by the fact that implantation of low doses of them into a 3.5‐mm diameter parietal bone defect in a biomaterial based on hydroxyapatite (HA), collagen, and polyethylene glycol hydrogel promotes the formation of new bone tissue within 5 weeks, which even in senile mice (18–22 months) covers the central part of the defect by 85%.

Today, bone autograft remains a safe, effective optimizer and gold standard for the repair of flat skull bone defects. Due to some known disadvantages, namely the need for additional surgery and the possible infection in the surgical area, premature resorption, and pain in the donor area, the number of operations using autografts is limited. The development of synthetic scaffolds is ongoing to replace lost bone tissue with the prospect of their biological properties approaching autologous bone [18]. The components of modern materials are biocompatible and biodegradable, both natural polymers (collagen, proteoglycans, alginate [Alg], and chitosan [CS]) and calcium phosphate (CaP) inorganic compounds (HA [19], tricalcium phosphate [TCP], brushite, dicalcium phosphate dihydrate [DCPD]). The physicochemical and biological characteristics of such composites (geometric shape, electrical conductivity, porosity, swelling degree, antibacterial, and antioxidant activity) demonstrate an optimizing effect on the healing of defects in the flat bones of the skull [20–24]. The healing of bone defects, the formation, and mineralization of regenerated bone tissue, and the integration of bone implants are also promoted by the additional use of vitamin D [25, 26]. The trace element Zn in some literary sources is now called “calcium of the 21st century,” has antibacterial properties, promotes osteoblast differentiation, and has an inhibitory effect on osteoclasts′ differentiation and resorption activity. Composite orthopedical implants made of Zn and HA have a regulated degradation rate, stimulating the process of bone formation and having antibacterial properties [27]. At the same time, experimental studies on small animals play a key role in the morphological assessment of the comparative effect of bone–plastic scaffolds on the healing of defects in flat skull bones with the aim of their further and potential application in human skull surgery [28].

This work is devoted to the problem of repairing defects in the bones of the cranial vault by implanting two different in‐structure osteoplastic materials, namely (a) based on the Alg matrix, modified with CaP microparticles (currently HA and Zn^2+^ ions) and (b) doped with CaP nanoparticles (currently DCPD) CS matrix. In previous works, we presented the physicochemical and medical–biological properties of such composites in the repair of femoral defects [29, 30]. As already noted, scientific literature, in most cases, indicates weak regenerative properties of the cranial vault bones. Therefore, this work is once again intended to confirm or refute the hypothesis of a positive effect of the presented osteoplastic materials on the dynamics of healing of cranial vault injuries. The study is based on computed tomography (СТ), as the most relevant and informative among x‐ray methods. CT is based on the study of tissue density, which is determined by the varying degree of absorption of x‐ray radiation by them. The CT method allows us to investigate the regenerative potential of these two biocomposites and compare their effectiveness with control samples in which healing occurs under the influence of a blood clot. Considering the effect of vitamin D on the healing of skull vault defects, we provide a detailed description of the cholecalciferol VitD3 release kinetics from the volume of biocomposites.

## 2. Materials and Methods (or Methods)

### 2.1. Experimental Design

This article continues the series of works on the subject and is devoted to assessing the regenerative potential of two composite materials, namely Alg_CaP_Zn_D3 and CS_CaP_D3. The detailed synthesis method and physical–chemical characterization of these composites are given in our previous works [29–31], and a graphic diagram of the technological process is shown in Figure 1.

Figure 1Schematic representation of the technology for producing composite materials: (a) Alg_CaP_Zn_D3 and (b) CS_CaP_D3.

**Figure:**
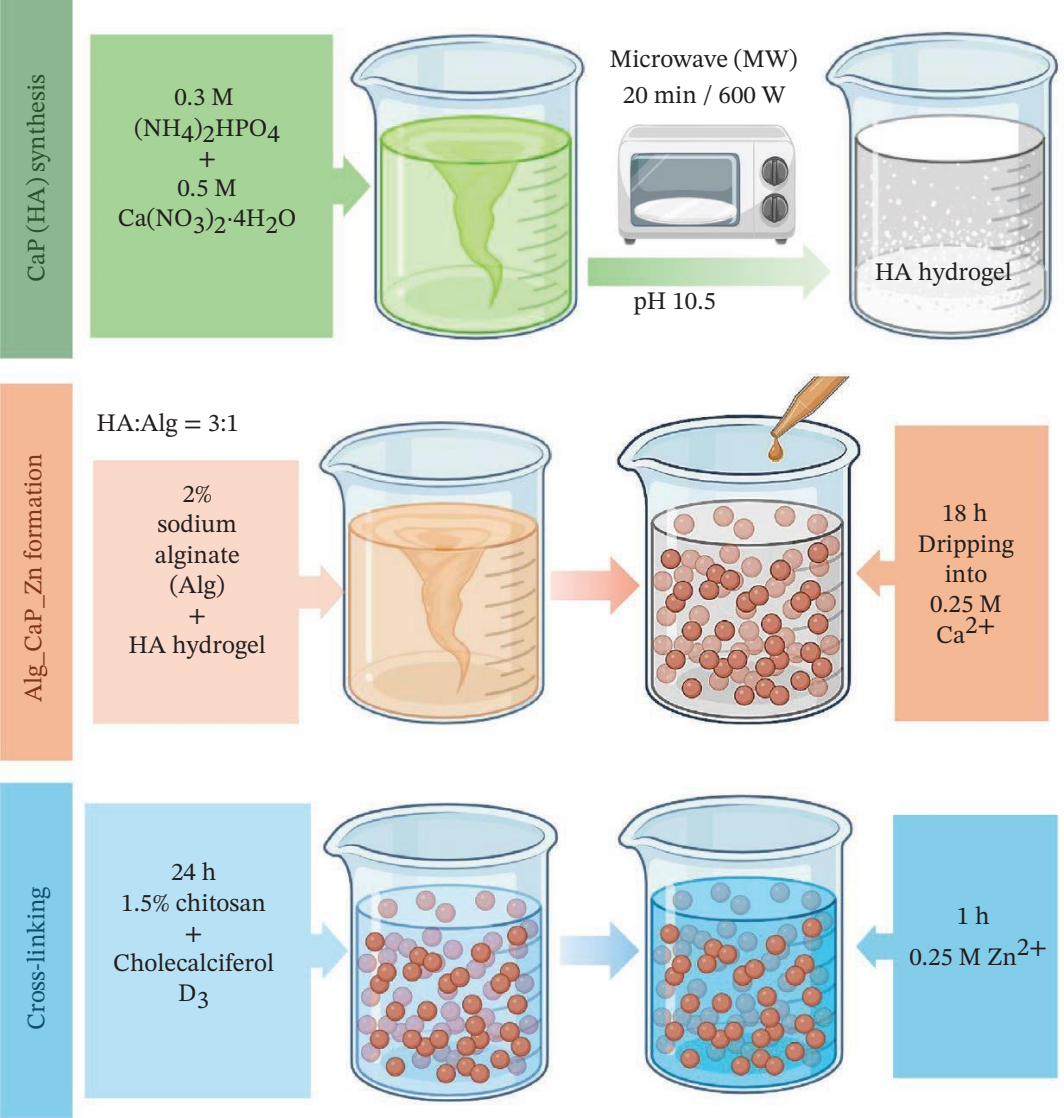
(a)

**Figure:**
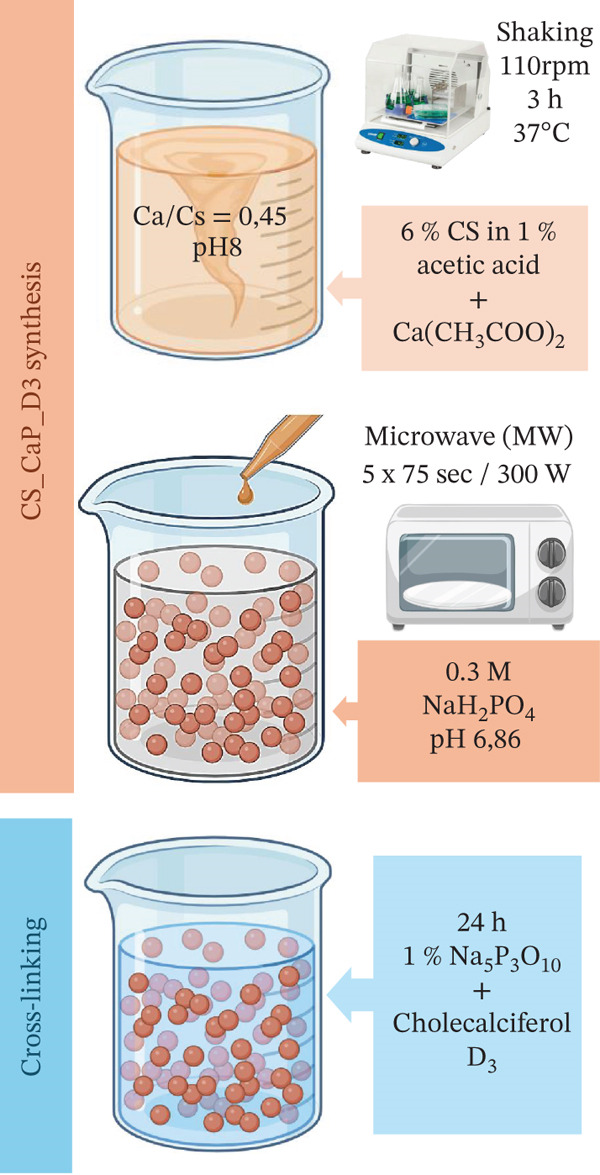
(b)

Briefly, matrices from Alg (a derivative of brown seaweed) and CS (a derivative of chitin, a component of crustacean shells) were used as scaffolds. Alg is an anionic polymer with the functional group СОО‐, and CS is a natural cation exchanger with a reactive amino group protonated in an acidic environment to NH_3_^+^.

When obtaining the Alg_CaP_Zn_D3 composite, the CaP component (currently nanostructured HA [Ca/P = 1.66]) was previously synthesized under the action of microwave irradiation (MW). It was combined in an ultrasonic disperser with a 2% solution of Alg (E401, Mm15.0 kDa, China) in the ratio CaP: Alg = 3 : 1 (in terms of dry matter) with subsequent formation of beads in a 0.25‐M CaCl_2_ solution. To give the beads mechanical stability, they were cross‐linked for 24 h in 1.5% CS (Mm 300 kDa, Acros organics, United States) and saturated with a solution of 0.25 mg/mL Vitamin D3 (commercially available pharmaceutical cholecalciferol VitD3). Zn ions were added from a solution of 0.25 M ZnSO_4_ × 7H_2_O. The washed and dried composite in beads with a 1–2‐mm diameter at 37°C was hereinafter called Alg_CaP_Zn_D3. The CaP phase content in the composite is 69%, the degree of swelling is 70.5%, Young′s modulus is 530 MPa, and the electrical conductivity is 0.1757 S/m [29].

When obtaining the CS_СаР_D3 composite, the synchronous synthesis method was used when the formation of the CS matrix and its simultaneous mineralization in situ by the formed СаР component under the action of microwave irradiation (300 W) for 75 s (5 × 15 s). The СаР component was identified by x‐ray diffraction as brushite (DCPD) with a *C* *a*/*P* = 1.0. Mechanical stability of the composite was provided in a 1% solution of sodium tripolyphosphate with added сholecalciferol VitD3 (0.25 mg/mL) for 24 h. Thus, CS_СаР_D3 beads of indefinite shape with dimensions of the larger side from 0.3 to 1.5 mm with a СаР phase content of about 24% and a swelling degree of 68% were obtained. Young′s modulus is 780 MPa, and electrical conductivity is 0.1707 S/m.

The appearance of the experimental beads is shown in Figure 2a,b.

Figure 2Electronic scanograms: (a) Alg_CaP_Zn_D3 size 1.18–1.55 mm and (b) CS_CaP_D3 size 1.12–1.49 mm. Magnification X 50.

**Figure:**
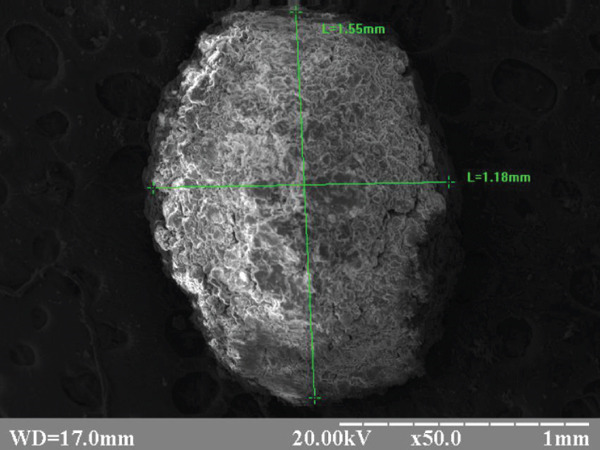
(a)

**Figure:**
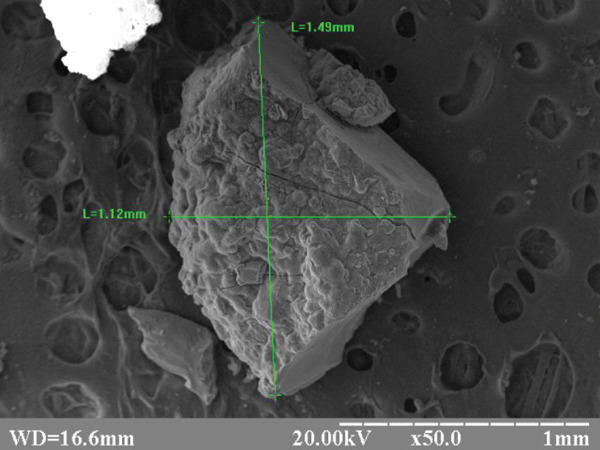
(b)

### 2.2. In Vivo in Rat Model Study

All experiments involving laboratory animals were conducted at Sumy State University (SSU) after approval by the Commission for Bioethics in Experimental and Clinical Research of SSU (Protocol No. 1/03 of 17.03.2025) and meet the requirements of the Law of Ukraine “On Medicinal Products” (1996, Articles 7, 8, and 12); ICH GCP (2008), GLP (2002) documents; requirements and standard provisions of the Order of the Ministry of Health of Ukraine dated September 23, 2009 No. 690 “On Approval of the Procedure for Conducting Clinical Trials of Medicinal Products and Examination of Clinical Trial Materials and the Standard Provision on the Ethics Commission.” Thirty‐six white laboratory rats were purchased. After a week of optimization in vivarium conditions, the rats were randomly divided into groups. To avoid potential bias in the selection of animals and variability in bone regeneration, we conducted an experimental study on rats of the same line (Wistar), same age (6 months), same sex (males), and same weight (200 ± 10 g). When taking the animals for the experimental study, we took into account their motor activity and the condition of the coat. If deviations in these parameters were found, the animals were not included in the experiment.

Then, according to the approved surgical protocol, an experimental defect was created in the parietal bone of the skull to the dura mater with subsequent implantation of Alg_CaP_Zn_D3 and CS_СаР_D3 composites into the defect cavity. The experimental bone defect was of the same size (2 mm) for all animals. Its application was performed under aseptic conditions and under anesthesia. Bone regeneration in all animals was studied at the same time (30th, 90th, 140th, and 200th day).

Before surgery, the rats were anesthetized by intramuscular injection of ketamine (50 mg/kg), xylazine (8 mg/kg), and acepromazine (4 mg/kg). In the area of the crown of the head, the hair was first cut off, and the skin was treated with a 3% alcoholic solution of iodine. A 1.5‐cm longitudinal incision was then made through the hairless skin and fascia, which were retracted to expose the parietal bone. To create the bone defect, a dental drill and a bur of uniform diameter (2 mm) were used for all animals. In order to avoid thermal damage to the edges of the defect and minimize unwanted hand movements during the procedure, the defect was created at low rotational speed with continuous irrigation using 0.9% sodium chloride solution. The surgical manipulation was performed very carefully so as not to damage the dura mater, as it plays an important role in the healing of defects in the bones of the cranial vault [28]. In the first control group, the bone defect was left to heal under a blood clot; in the second experimental group, it was filled with Alg_CaP_Zn_D3, and in the third, CS_CaP_D3. The surgical wound was closed with polyglactin sutures. For analgesia, rats were given buprenorphine (0.3 mg/kg) subcutaneously every 12 h for 2 days after surgery. On the 30th, 90th, 140th, and 200th days after scaffold implantation, the animals were not euthanized. The rats were anesthetized (50‐mg/kg ketamine) and examined using a 16‐slice spiral СТ scanner “TOSHIBA Activion” (Toshiba, Japan).

Both before and after surgery, the experimental animals were kept in a vivarium in comfortable temperature conditions (air temperature of about 21°C); they were fed complete rat food with supplementary food (eggs, vegetables, and fruits). The animals consumed water from drinking bowls. The cells were cleaned daily.

Using СТ, the effect of the studied scaffolds on the possibility of closing the defect with dense, mineralized bone tissue was evaluated, and a three‐dimensional reconstruction of the injury site was performed, measuring the optical density of the regenerated bone tissue in Hounsfield units.

On the 30th, 90th, 140th, and 200th day after implantation of bone grafting materials, the absolute optical density (AOD) of the biomaterial implantation site and the adjacent cortical layer of the mother bone was measured in Hounsfield units.

The obtained indicators of the AOD of the regenerate made it possible to calculate the relative optical density of the regenerate (ROD) in comparison with the absolute optical density of the adjacent to the defect site of the mother bone (AODMB):

ROD=AODx100%/AODMBHU.

### 2.3. Drug Release

The release of cholecalciferol (vitamin D3) was studied using an Agilent 1200 chromatography system, Zorbax SB‐C18 column (4.6 × 150 mm, 5 *μ*m). Mobile phase: acetonitrile–water in a ratio of 90:10. Detection was performed on a UV–Vis Abs detector at a wavelength of *λ* = 250 nm. The isocratic method was used with an elution rate of 1 mL/min at room temperature.

### 2.4. Statistical Analysis

Statistical analysis was performed using GraphPad Prism 5.0 software. The numerical data obtained were expressed as mean (± standard error of the mean [SEM]). The significance of differences between the compared indicators was assessed using Student′s *t*‐test. ∗, *p* < 0.05; ∗∗, *p* < 0.005; ∗∗∗, *p* < 0.0005; and ∗∗∗∗, *p* < 0.00005 were considered statistically significant, and ns as statistically nonsignificant.

## 3. Results

**Figure 3:**
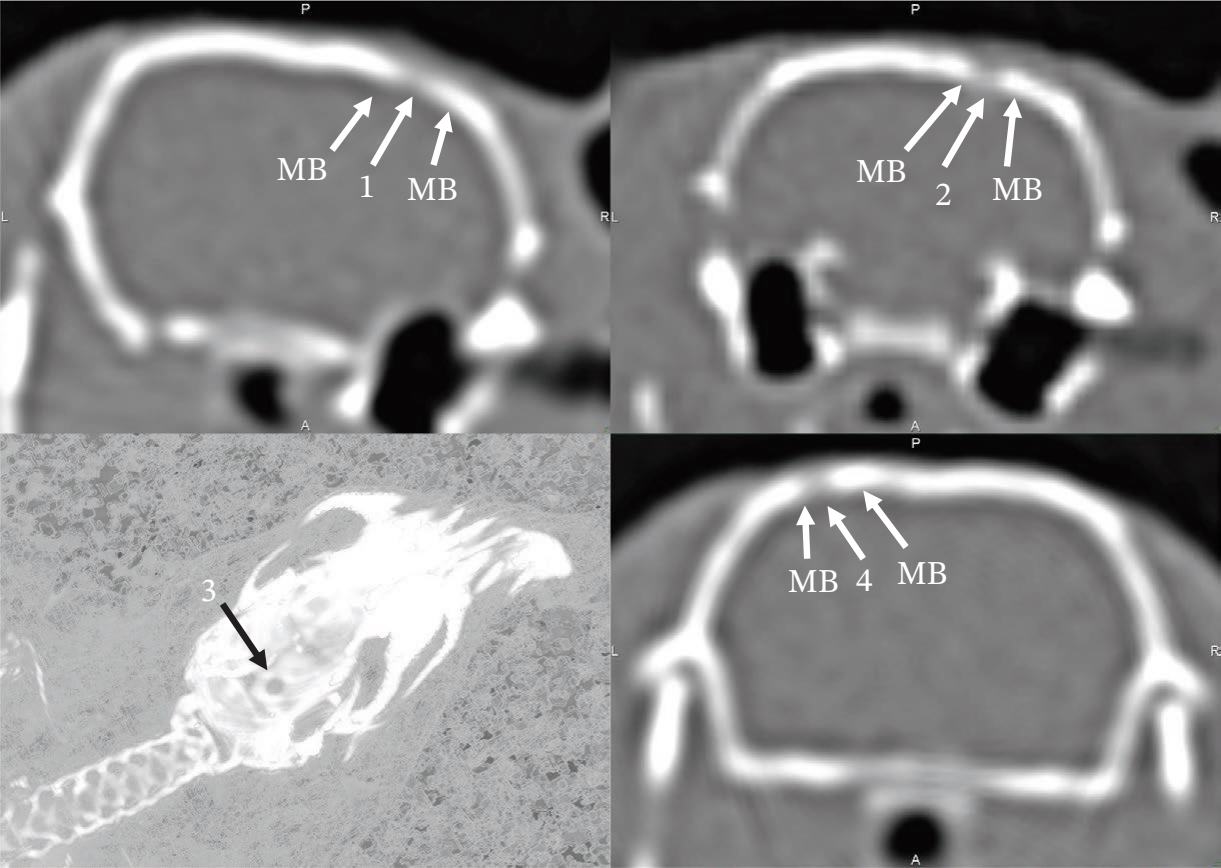
Computed tomograms of the rat skull on the 30th day after implantation of biomaterials into the parietal bone defect area. Injury area in animals of the control group (1) and with implanted Alg_CaP_Zn_D3, section (2), and 3D model (3); with CS_CaP_D3 (4).

**Figure 4:**
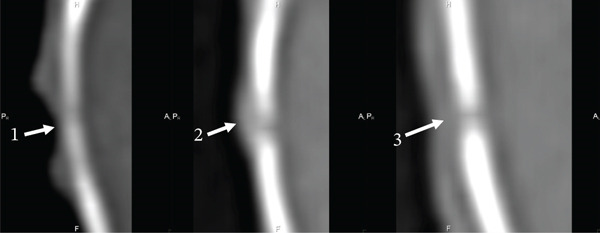
Computed tomograms of the rats′ parietal bones in lateral projection on the 30th day after implantation. Injury area in animals of the control group (1), with implanted Alg_CaP_Zn_D3 (2), and CS_СаР_D3 (3).

On the 30th day of the experiment, all the studied animals had a round defect with precise contours in the parietal bone area (Figures 3 and 4). Such a radiological picture was possible because in the animals of the first control, second Alg_CaP_Zn_D3, and third CS_CaP_D3 groups, the optical density of the maternal bone (MB) (1342.93 ± 28 HU, 1192.88 ± 24 HU, 1595.53 ± 29 HU) was significantly higher than in the injury area (684.96 ± 5.7 HU, 592.16 ± 53 HU, 840.95 ± 29 HU), respectively.

Thus, on Day 30, low optical density was observed both in the area of CS_CaP_D3 implantation (with a low CaP content of 24%) and in the area implanted with Alg_CaP_Zn_D3, despite its high CaP phase content (69%). This can be explained by the fact that the CaPs synthesized in this study at low temperature (37°C) were nanocrystalline, lacking a defined crystalline structure and, consequently, exhibiting low radiopacity during the early stages of implantation. This observation is consistent with previous studies on the early phases of HA synthesis from precipitates [32, 33]. The subsequent increase in optical density indicates radiological signs of bone tissue formation and maturation within the regenerate.

**Figure 5:**
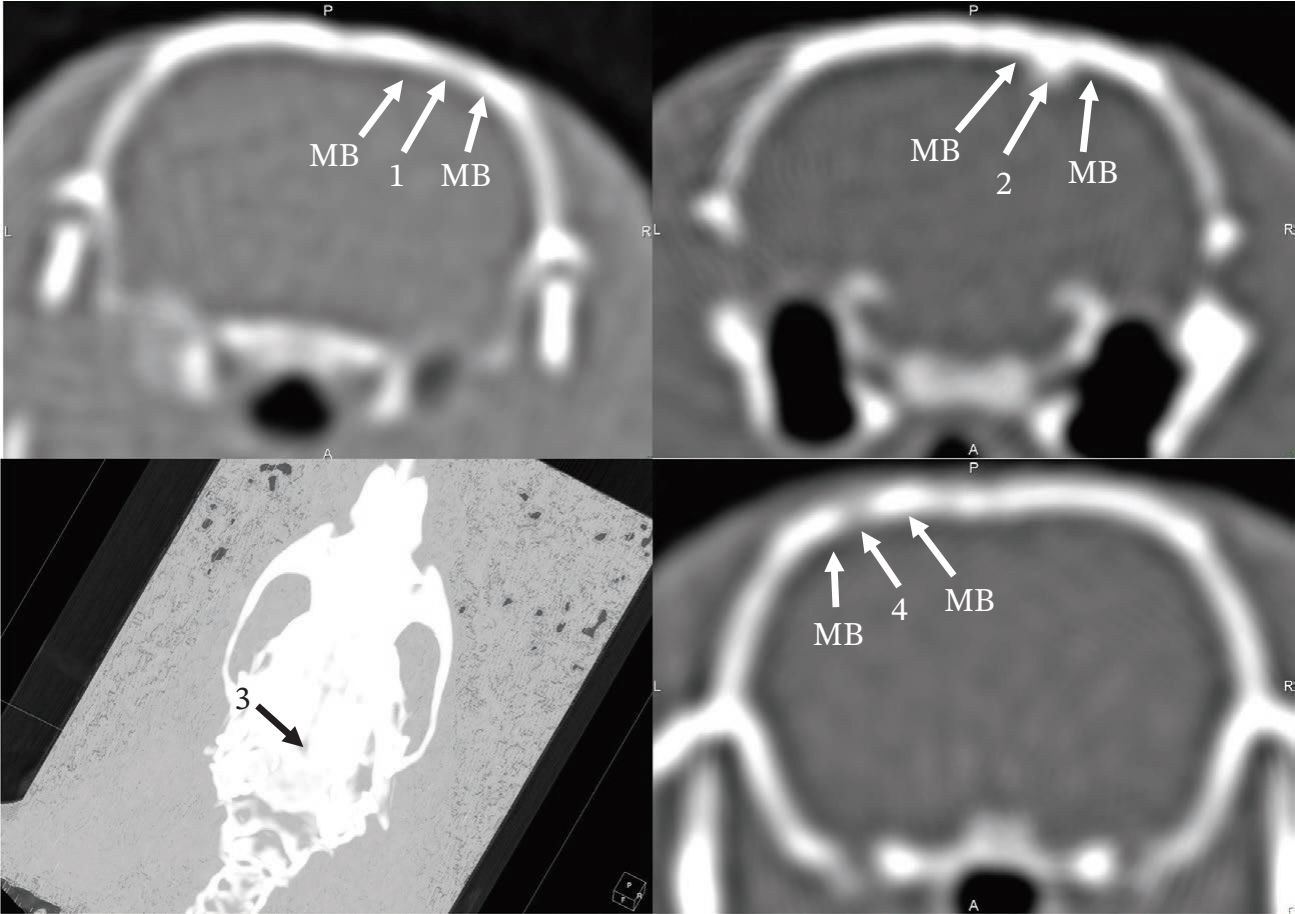
Computed tomograms of the rat skull on the 90th day after biomaterials implantation. Injury area in animals of the control group (1) and with implanted Alg_CaP_Zn_D3: Section (2), 3D model (3), and with CS_CaP_D3 (4).

**Figure 6:**
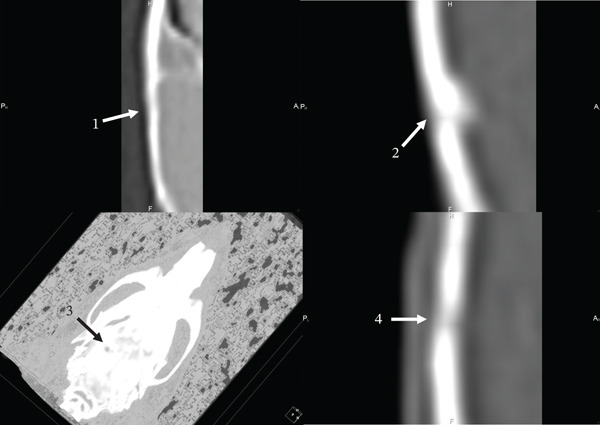
Computed tomograms of the parietal bones of rats in lateral projection on the 90th day after the defect and biomaterials implantation. Injury area in animals of the control group (1), with implanted biomaterials Alg_CaP_Zn_D3 (2), and CS_CaP_D3: 3D model (3), section (4).

**Figure 7:**
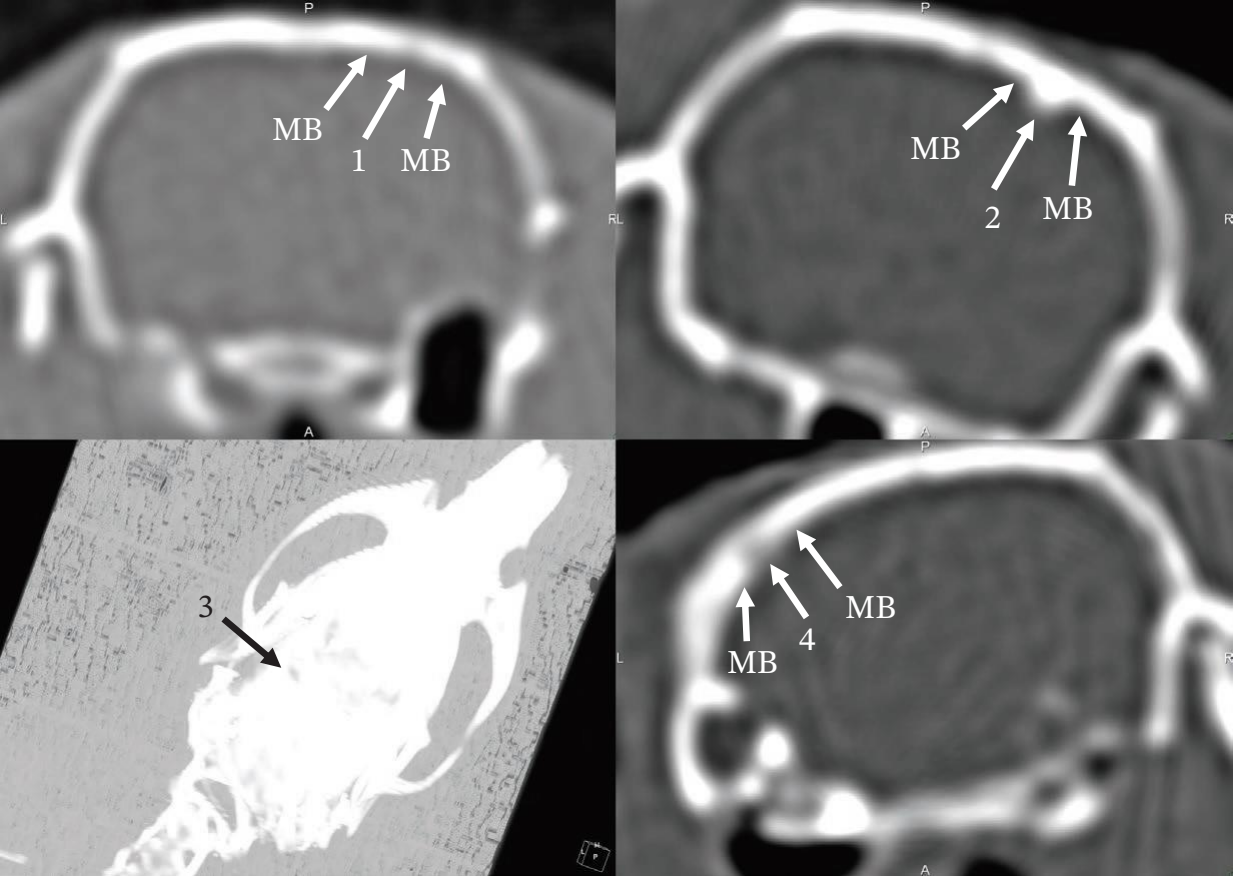
Computed tomograms of the rat skull on the 140th day after creating a defect in the parietal bone and implantation of biomaterials. Injury site in animals of the control group (1) and with implanted Alg_CaP_Zn_D3 (2) and CS_CaP_D3: 3D model (3), section (4).

**Figure 8:**
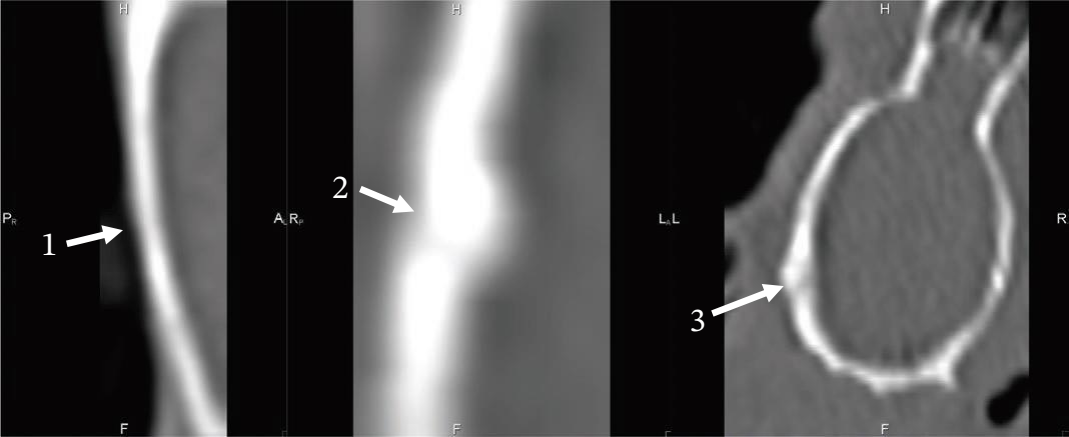
Computed tomograms of the parietal bones of rats in lateral projection on the 140th day after the defect and implantation of biomaterials. Injury area in animals of the control group (1) and with implanted biomaterials Alg_CaP_Zn_D3 (2), CS_СаР_D3 (3).

**Figure 9:**
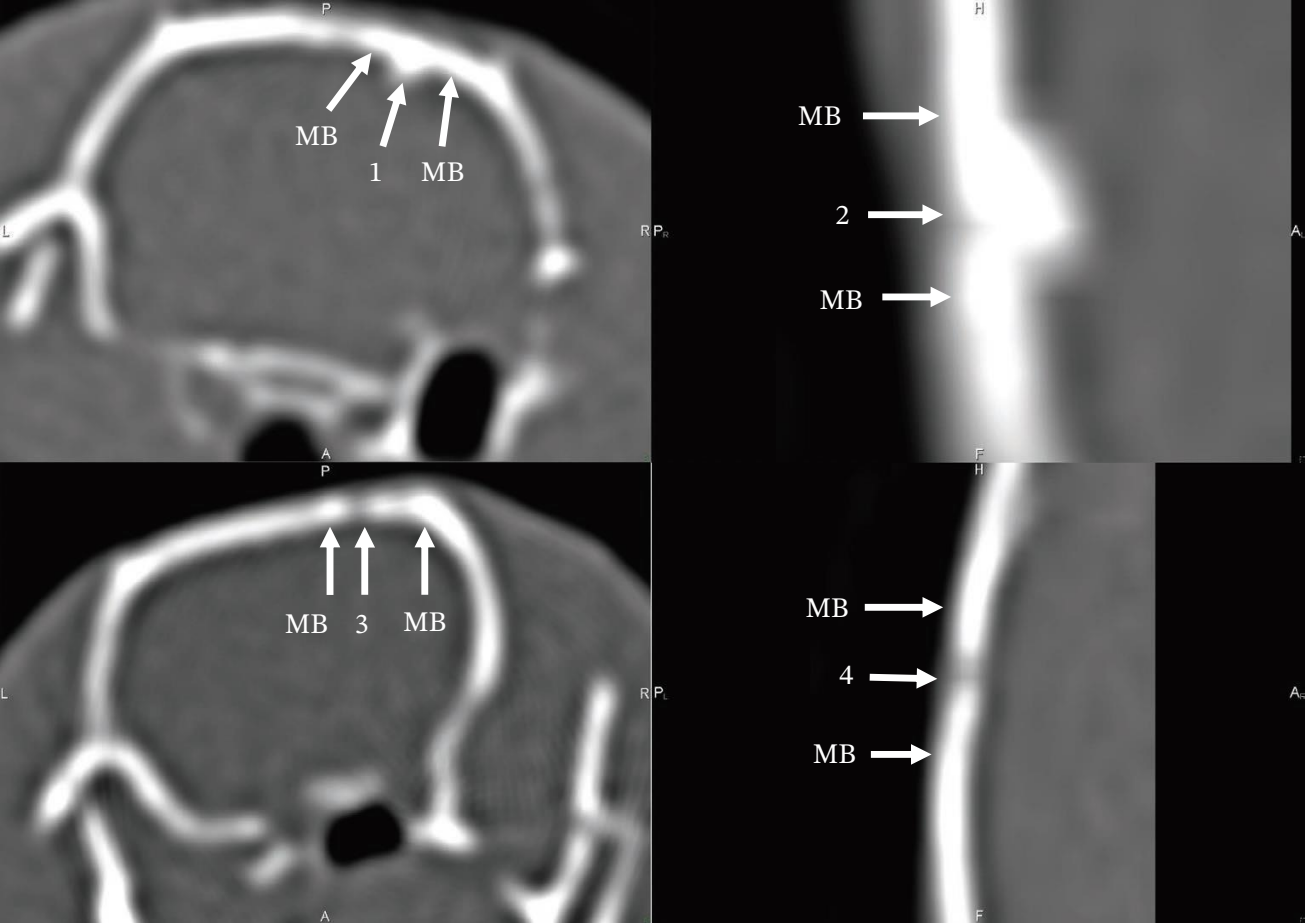
Computed tomograms of the rat skull on the 200th day after the defect in the parietal bone. The area of implantation of the Alg_CaP_Zn_D3 in the front (1) and lateral (2) projections. The area of injury with implanted CS_CaP_D3 (3) and in animals of the Control group, where the defect healed under a blood clot (4).

**Figure 10:**
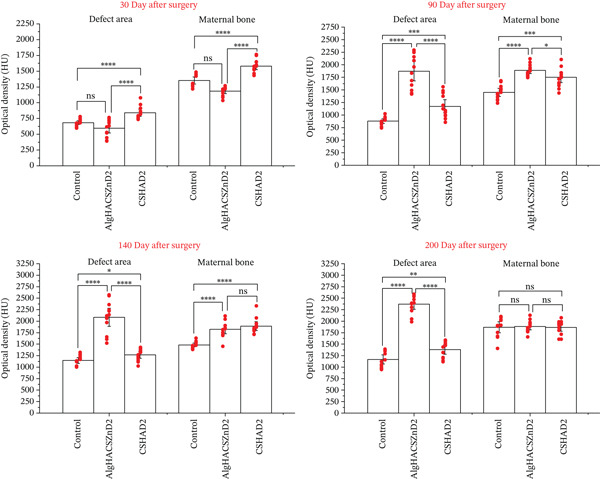
Data of variational–statistical processing (*M* ± *m*) of AOD of the defect area and the adjacent MB in Hounsfield units (*n* = 12).

On the 90th day of the experiment, in animals of the control group, the AOD increased by 27.94% and amounted to 876.36 ± 17 HU. Since the studied indicator was 1.64 times lower than in the adjacent MB (1438.01 ± 28 HU), the defect area was well visualized on CT (Figures 5 [1] and 6 [1]). The CS_CaP_D3 implantation site was also well visualized both on CT (Figure 5 [4]) and on the 3D skull model (Figure 6 [3]) at this time of the experiment, since its AOD (1138.34 ± 36 HU) although increased 1.35 times compared with the previous time, was 1.56 times lower than that of the adjacent MB (1786.72 ± 37 HU) (Figures 5 [4] and 6 [3, 4]). The calculations confirm the presence of a significant statistical difference between the optical density of the defect zone for all samples (Figures 7, 8 and 9). At the same time, the AOD of CF in the implantation sites of Alg_CaP_Zn_D3 and CS_CaP_D3 differ little from each other (Figure 10, 90 days).

In the area of Alg_CaP_Zn_D3 implantation, the AOD (1894.7 ± 62 HU) increased 3.19 times compared with the 30th day of the experiment and no longer differed from the similar indicator of the MB (1872.77 ± 21 HU). At the same time, the area of the past injury was not visually traced on the 3D skull model (Figure 5 [3]). On CT, it was detected only due to the thickening of the endosteum and a slight depression on the side of the periosteum, as well as an area (gap) of reduced density, which was located on one side of the MB at the level of implantation of the bone–plastic material (Figures 5 [2] and 6 [2]).

On the 140th day of the experiment, the size of the bone defect in the control group animals significantly decreased (Figure 7 [1]). The AOD of the latter was 1150.9 ± 22 HU, which was 1.36 times higher than on the 90th day. At the same time, the area of the previous injury in the control group animals was still visualized on the CT because its AOD was 1.28 times lower than the similar indicator of the MB (1478.79 ± 19 HU) (Figure 7 [1], Figure 8 [1]), Figure 10.

In the area of Alg_CaP_Zn_D3 implantation, the AOD increased 1.13 times (*p* < 0.05) compared with the previous observation period and was 2144.72 ± 61 HU, which exceeded the AOD of the MB (1815.56 ± 34 HU) by 1.18 times; however, due to the thickening from the endosteal side at the level of Alg_CaP_Zn_D3 implantation (Figure 7 [2]) and the area of reduced density between the regenerate and the edge of the MB (Figure 8 [2]), the location of the past injury could be identified on the CT.

In the area of CS_CaP_D3 implantation, there is an increase in AOD (1271 ± 23 HU) by 11.65% compared with the 90th day of the experiment. However, the injury area was well visualized both in 3D models (Figure 7 [3]) and on CT (Figure 7[4]) since the AOD of the MB (1889.26 ± 36 HU) significantly exceeded the similar indicator of the area of CS_CaP_D3 implantation.

Since the animals implanted with Alg_CaP_Zn_D3 showed the most significant potential for regenerating the parietal bone defect, we continued the further study. On the 200th day after implantation, it was found that the AOD of the Alg_CaP_Zn_D3 implantation site increased to 2387.38 ± 47 HU, which exceeded the optical density of the MB (1907.57 ± 36 HU), the injury site of the control animals (1234 ± 60 HU) and the CS_CaP_D3 implantation site (1383.67 ± 51 HU) by 1.25 (*p* < 0.05), 1.93 (*p* < 0.05) and 1.72 times (*p* < 0.05), respectively. However, in animals with implanted Alg_CaP_Zn_D3, an endosteal callus and a slight depression on the periosteum side were still visually observed, and the area of reduced density between the regenerate and the edge of the MB was no longer determined (Figure 9).

The summarized data on the optical density of the defect area and the adjacent MB during the specified study periods are shown in Figure 10.

As we can see, at the initial stage there is no significant statistical difference between the indicators of the control and the *Alg_CaP_Zn_D3* group. However, after 90 days, the healing dynamics take an opposite turn. The defect zone in the *Alg_CaP_Zn_D3* group has already reached the level of the MB. Meanwhile, in the *CS_CaP_D3* group, the rate of MB regeneration is nearly identical to that of the control group. It is important to note that after 200 days postsurgery, the density values for the MB show no significant differences among all samples, indicating that metabolic processes in the damaged area are no longer as active. At the same time, the AOD in the Alg_CaP_Zn_D3 implantation zone significantly exceeds the values for both the other two samples and the AOD of the MB.

### 3.1. Drug Release

**Figure 11:**
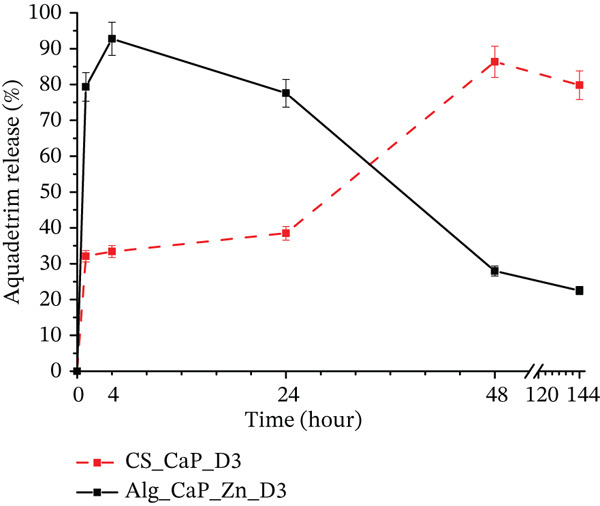
Cumulative release of cholecalciferol VitD3 from Alg_CaP_Zn_D3 and CS_СаР_D3 composites (*n* = 3, *p* ≤ 0.05).

This section is devoted to the in vitro study of the kinetics of VitD3 release from experimental biocomposites and the effect of matrix components on this process (Figure 11). As mentioned, cholecalciferol VitD3 was added to the composites by saturation from its alcohol solution with a 0.25‐mg/mL concentration. The results of VitD3 release into phosphate‐buffered saline (PBS) obtained by HPLC showed that within 48 h, adsorbed cholecalciferol was almost completely released from both samples, namely 93% from Alg_CaP_Zn_D3 and 80% from CS_CaP_D3 (Figure 10). However, the release kinetics differed significantly. This difference may be due to the different interactions between vitamin D3 and the components of biomaterials. For example, Alg_CaP_Zn_D3 contains about 70% CaP (HA), which is a good adsorbent. In addition, the desorption of the drug in saline occurs due to ion exchange during the swelling of the matrix; vice versa, CS_CaP_D3 contains about 24% CaP component.

The Alg_CaP_Zn_D3 sample demonstrates a “burst release” during the first 4 h of the study, resulting from сholecalciferol VitD3 desorption from the matrix due to ion exchange when composite swells in PBS solution. The maximum release in the case of CS_CaP_D3 is observed after 48 h, whereas the release rate is not uniform. Thus, during the first 4 h, only 30% of the surface‐adsorbed drug is released (vs. 80% from the Alg‐containing matrix), followed by a slow release of up to 38% after 24 h. During the second day, the drug is released more rapidly and almost completely.

Thus, the CS_CaP_D3 composite does not release excessive amounts of cholecalciferol VitD3 during the first hours and prolongs the drug release up to 48 h. On the contrary, in the case of Alg_CaP_Zn_D3, we observe a “burst release” of the VitD3 within 4 h.

## 4. Discussion and Conclusion

An experimental study established that on the 30th day after implantation in animals of all experimental groups, the AOD of the parietal bone defect area was significantly lower than that of the adjacent MB (Figure 10). Despite the fact that the synthesized scaffolds contained HA, low optical density was observed both in the implantation area of CS_CaP_D3 with a low CaP content (24%) and in the implantation area of Alg_CaP_Zn_D3 with a high CaP phase content in the composite (69%). This can be explained by the fact that the CaPs synthesized in this study at a low temperature (37°C) are nanocrystalline and amorphous, lacking a crystalline structure and, consequently, the radiopacity typically observed during the initial stages of implantation [32]. As a result, the area of implantation of biocomposites on CT was well traced but limited by the precise contours of the MB. At the same time, the AOD of the injury area of the animals of the control group, the area of implantation of Alg_CaP_Zn_D3 was almost the same, but significantly lower than in the defect area of rats with implanted bone–plastic material CS_CaP_D3. Thus, at this time of the experiment, no radiological signs of defect closure with mineralized bone tissue were observed in any group of animals.

Volkov et al. also reported that after 28 days, the healing of a defect (8 mm in diameter) in the parietal bone of rats occurs only by 10%. However, in the area of implantation of bone–plastic material based on poly(3‐hydroxybutyrate), HA, Alg, and mesenchymal stem cells (poly(3‐hydroxybutyrate)/HA/Alg scaffolds seeded with mesenchymal stem cells, PHB/HA/ALG/MSC), almost complete closure of the defect with bone tissue (up to 98.3%) is observed. It should be noted that in this work, scientists do not provide digital indicators of the density of the injury site. Still, visually, the biomaterial implantation site′s density is significantly lower than that of intact bone on the CT, as shown in the article [24]. Song et al. used micro‐CT to establish that implantation of chitosan, fibroin‐hydroxyapatite (CFB‐HAP) membranes into a defect (8 mm) in the middle of the skull vault of male Sprague–Dawley rats after 8 weeks contributes to an increase in the mineral density of the injury site (0.83 ± 0.13 mg · mL^−1^) and bone volume (8.70 ± 3.57  mm^3^) compared with the control group (0.67 ± 0.08 mg · mL^−1^ and 4.78 ± 2.03  mm^3^). However, complete recovery of the bone defect does not occur within 8 weeks [34].

After 3 months of experimentation, we also observed a tendency to increase the AOD of the injury site in animals of all groups. The minimum value of the studied indicator was in animals of the control group and in the area of implantation of CS_CaP_D3. In animals of these groups, the given indicators did not even approach the level of the MB. Therefore, the area of the past injury was well visualized on the CT. At the same time, the AOD of the Alg_CaP_Zn_D3 implantation site increased 3.19 times (*p* < 0.05) compared with the previous period, exceeding the injury site of the control group animals and the CS_CaP_D3 implantation site by 2.16 (*p* < 0.05) and 1.66 times (*p* < 0.05), respectively, and equalled the similar indicator of the MB. However, due to the presence of a pronounced endosteal callus, a slight depression on the periosteum and a low‐density area between the regenerate and the edge of the MB adjacent to the defect, the Alg_CaP_Zn_D3 implantation zone could be visualized on CT.

In the work of He et al., the effect of a bone–plastic material based on CS, Alg, and HA was investigated. It was found that a biocomposite scaffold 12 weeks after implantation into an 8‐mm diameter defect in the skull of 8‐week‐old male rats contributed to an increase in the mineral density of the injury site almost twice as much as in the control group. Despite this, the bone defect did not heal after 12 weeks with the implanted biocomposite material [35]. Im et al., using quantitative evaluation of micro‐CT images of reparative regeneration of a 4‐mm bone defect in the right parietal bones of 60‐day‐old nude mice, also demonstrated that after 8 weeks, the defect healed by less than 10% in the control group, and by 80% in the group implanted with HA/*β*‐TCP [36]. It should be noted that in those groups of animals that were implanted with scaffolds based on CS, Alg,, and HA with the addition of bone morphogenetic protein‐2 and mesenchymal stem cells, the defect was closed entirely by bone tissue [35], and in the area of implantation of HA/*β*‐TCP with mesenchymal stem cells, by 90% [37]. In our experiment, after 140 days, a noticeable decrease in the defect size was observed in the animals of the control group, and the relative OD of the injury area was 77.76%, whereas in the area of CS_СаР_D3 implantation was 67.27%. Therefore, the visualization of the past injury in the animals of these groups was evident. The lowest AOD was observed in the animals of the control group (1150.9 ± 22 HU) and in the area of CS_СаР_D3 implantation (1271 ± 23 HU), whereas the OD of the Alg_CaP_Zn_D3 area was 2144.72 ± 61 HU. The latter indicator was not only the highest among all the animals studied but also exceeded the AOD of the MB. Due to this, the relative optical density of the Alg_CaP_Zn_D3 implantation site was more significant than 100%, which indicates the repair of the bone defect. However, the location of the past injury could still be visualized by the existing endosteal callus, a slight depression on the periosteal side and a reduced density area between the regenerate and the edge of the MB. In our opinion, the reason for the formation of the endosteal callus is the larger size of the implanted composite bead compared with the thickness of the parietal bone, so part of the bead is in the epidural space. At the same time, on the 200th day of the experiment, the reduced density area between the regenerate and the edge of the MB disappears. Still, the endosteal callus and a slight depression on the periosteal side remain. The AOD of the Alg_CaP_Zn_D3 implantation site over 200 days of the experiment exceeded by 1.25 times (*p* < 0.05) the similar indicator of the MB, which, according to the x‐ray examination, indicates the recovery of the bone defect. However, the AOD in the animals of the control group and the area of CS_СаР_D3 implantation was still significantly lower than in the MB adjacent to the injury site. Due to this, the injury site in the given animals was well visualized on the CT.

The role of vitamin D3 in the healing after bone damage is currently a widely studied topic, as previously all attention was paid to preventing damage rather than treating it [37]. Its pharmacological effect is to regulate the exchange between calcium and phosphate ions. Calcium mobilization, which involves vitamin D3, is important for the attachment and proliferation of osteoblasts on the surface of composite materials and bone formation. Vitamin D3 activates the uptake of phosphate ions by bone tissue and enhances the ossification process [38]. Recent human clinical trials have shown a low effect of vitamin D3 on bone regeneration [37]. It is expected that the combined use of vitamin‐rich biopolymer–apatite composites will show improved results. Summarizing the results of the HPLC study, it can be assumed that in the case of Alg_CaP_Zn_D3, cholecalciferol VitD3 release is controlled to a greater extent by the CaP component and matrix swelling (higher degree of swelling compared with CS_CaP_D3). Stronger electrostatic bonds between CS and cholecalciferol VitD3 molecules and higher mechanical stability of CS_CaP_D3 composite at pH 7.4 are the keys to the kinetics of cholecalciferol VitD3 release from this matrix. Thus, rapid release within 1–2 days can provide the necessary concentration of cholecalciferol in the area of injury to activate the mechanisms of calcium–phosphorus metabolism. A similar trend is observed in other studies on the release of cholecalciferol VitD3 from polymer matrices, but the release time is lower than that demonstrated in this article [39, 40].

This work continues the cycle of our studies on comparing the regenerative potential of two composite biomaterials in treating defects of femoral bone tissue and bones of the cranial vault of experimental animals. Both osteoplastic materials show the potential to optimize reparative osteogenesis in an experimental defect of the flat skull bone, whereas the regenerative potential of CS_CaP_D3 is lower than that of Alg_CaP_Zn_D3.

## Funding

This study was supported by the National Research Foundation of Ukraine, (10.13039/100018227) (0122U001154).

## Ethics Statement

All experiments involving laboratory animals were conducted at Sumy State University (SSU) after approval by the Commission for Bioethics in Experimental and Clinical Research of SSU (Protocol No. 1/6 of 30.06.2023) and meet the requirements of the Law of Ukraine “On Medicinal Products” (1996, Articles 7, 8, and 12); ICH GCP (2008), GLP (2002) documents; requirements and standard provisions of the Order of the Ministry of Health of Ukraine dated September 23, 2009 No. 690 “On Approval of the Procedure for Conducting Clinical Trials of Medicinal Products and Examination of Clinical Trial Materials and the Standard Provision on the Ethics Commission.”

## Conflicts of Interest

The authors declare no conflicts of interest.

## Data Availability

Data are available on request from the authors.
